# Abnormalities of fixation, saccade and pursuit in posterior cortical atrophy

**DOI:** 10.1093/brain/awv103

**Published:** 2015-04-20

**Authors:** Timothy J. Shakespeare, Diego Kaski, Keir X. X. Yong, Ross W. Paterson, Catherine F. Slattery, Natalie S. Ryan, Jonathan M. Schott, Sebastian J. Crutch

**Affiliations:** 1 Dementia Research Centre, Department of Neurodegenerative Disease, Institute of Neurology, University College London, London, UK; 2 Division of Brain Sciences, Imperial College London, Charing Cross Hospital, London UK

**Keywords:** Alzheimer’s disease, visual function, parietal lobe, agnosia, oculomotor

## Abstract

The clinico-neuroradiological syndrome posterior cortical atrophy is the cardinal ‘visual dementia’ and most common atypical Alzheimer’s disease phenotype, offering insights into mechanisms underlying clinical heterogeneity, pathological propagation and basic visual phenomena (e.g. visual crowding). Given the extensive attention paid to patients’ (higher order) perceptual function, it is surprising that there have been no systematic analyses of basic oculomotor function in this population. Here 20 patients with posterior cortical atrophy, 17 patients with typical Alzheimer’s disease and 22 healthy controls completed tests of fixation, saccade (including fixation/target gap and overlap conditions) and smooth pursuit eye movements using an infrared pupil-tracking system. Participants underwent detailed neuropsychological and neurological examinations, with a proportion also undertaking brain imaging and analysis of molecular pathology. In contrast to informal clinical evaluations of oculomotor dysfunction frequency (previous studies: 38%, current clinical examination: 33%), detailed eyetracking investigations revealed eye movement abnormalities in 80% of patients with posterior cortical atrophy (compared to 17% typical Alzheimer’s disease, 5% controls). The greatest differences between posterior cortical atrophy and typical Alzheimer’s disease were seen in saccadic performance. Patients with posterior cortical atrophy made significantly shorter saccades especially for distant targets. They also exhibited a significant exacerbation of the normal gap/overlap effect, consistent with ‘sticky fixation’. Time to reach saccadic targets was significantly associated with parietal and occipital cortical thickness measures. On fixation stability tasks, patients with typical Alzheimer’s disease showed more square wave jerks whose frequency was associated with lower cerebellar grey matter volume, while patients with posterior cortical atrophy showed large saccadic intrusions whose frequency correlated significantly with generalized reductions in cortical thickness. Patients with both posterior cortical atrophy and typical Alzheimer’s disease showed lower gain in smooth pursuit compared to controls. The current study establishes that eye movement abnormalities are near-ubiquitous in posterior cortical atrophy, and highlights multiple aspects of saccadic performance which distinguish posterior cortical atrophy from typical Alzheimer’s disease. We suggest the posterior cortical atrophy oculomotor profile (e.g. exacerbation of the saccadic gap/overlap effect, preserved saccadic velocity) reflects weak input from degraded occipito-parietal spatial representations of stimulus location into a superior collicular spatial map for eye movement regulation. This may indicate greater impairment of identification of oculomotor targets rather than generation of oculomotor movements. The results highlight the critical role of spatial attention and object identification but also precise stimulus localization in explaining the complex real world perception deficits observed in posterior cortical atrophy and many other patients with dementia-related visual impairment.

## Abstract (precis)

Oculomotor function in the ‘visual dementia’ posterior cortical atrophy (PCA) has received little attention. Shakespeare et al. report impairments in fixation, saccade and smooth pursuit in patients with PCA and typical Alzheimer’s disease, and suggest that oculomotor impairment should be considered a core feature of the PCA syndrome.

## Graphical abstract

**Figure:**
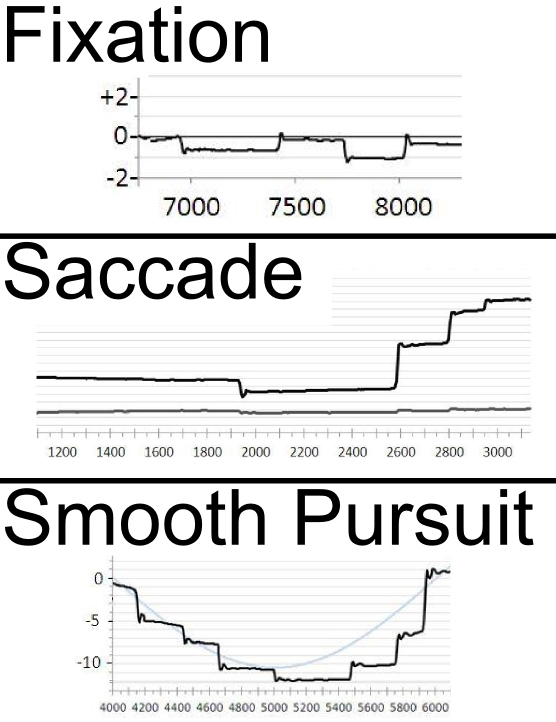
Oculomotor function in the ‘visual dementia’ posterior cortical atrophy (PCA) has received little attention. Shakespeare et al. report impairments in fixation, saccade and smooth pursuit in patients with PCA and typical Alzheimer’s disease, and suggest that oculomotor impairment should be considered a core feature of the PCA syndrome.

## Introduction

Posterior cortical atrophy (PCA) is a clinico-radiological syndrome characterized by insidious decline in visuoperceptual, visuospatial and other posterior cortical skills and atrophy of the parietal and occipital lobes (Benson *et al.*, 1988; Mendez *et al.*, 2002; Tang-Wai *et al.*, 2004; see Crutch *et al.*, 2012 for a review). The most common pathology in PCA is Alzheimer’s disease, and PCA is now recognized in Alzheimer’s disease diagnostic and research criteria as the most common atypical Alzheimer’s disease phenotype (McKhann *et al.*, 2011; Dubois *et al.*, 2014). However, the syndrome can also be caused by Lewy body disease and corticobasal degeneration (Hof *et al.*, 1990; Tang-Wai *et al.*, 2003; Renner *et al.*, 2004; Kouri *et al.*, 2011). PCA is typically an early-onset, sporadic condition (age 50–65 years) (Mendez *et al.*, 2002; Tang-Wai *et al.*, 2004; McMonagle *et al.*, 2006).

There is currently much greater discussion and examination of high-level object and space perception problems than more fundamental deficits of perceptual and oculomotor function. For example, impairment is widely reported on tasks of higher order perception (e.g. non-canonical object recognition, complex spatial analysis) which rely on cognitive processes associated with parietal and occipito-temporal mechanisms downstream in the visual system. By contrast, more basic visual functions (e.g. edge detection, form and motion coherence) which may underpin many such downstream deficits and which are mediated largely by upstream occipital mechanisms, have been largely overlooked (*cf.* Lehmann *et al.*, 2011*a*).

Even more notable by their absence are any systematic studies of oculomotor function in PCA. Previous studies have identified distinct oculomotor profiles differentiating dementia populations (frontotemporal lobar degeneration, corticobasal degeneration, progressive supranuclear palsy, and typical Alzheimer’s disease: Garbutt *et al.*, 2008; Boxer *et al.*, 2012; amyotrophic lateral sclerosis: Sharma *et al.*, 2011; Huntington’s disease: Golding *et al.*, 2006; Hicks *et al.*, 2008; progressive supranuclear palsy: Rohrer *et al.*, 2010) and associations between eye movements and higher order perceptual and spatial functions (Bak *et al.*, 2006), but the profile of oculomotor function in PCA has not yet been determined. Some clinical reports of PCA have detailed the frequency of symptoms such as oculomotor apraxia (e.g. 38% of 39 patients with PCA reported by Kas *et al.*, 2011; see also Mendez *et al.*, 2002; Nestor *et al.*, 2003; Tang-Wai *et al.*, 2004; McMonagle *et al.*, 2006) and ‘sticky fixation’ (a deficit in disengagement from a target; Delamont *et al.*, 1989), but estimates are largely derived from clinical examination or inferred from clinical history or impairments observed on other more complex tasks.

There are strong scientific and theoretical rationales for investigating oculomotor function in PCA. Patients with PCA (whether or not named as such) are frequently used in neuroscientific investigations of a host of visual processes such as visual crowding (Yong *et al.*, 2014), visual salience (Mannan *et al.*, 2009; Foulsham *et al.*, 2011), global/local processing and simultanagnosia (Graff-Radford *et al.*, 1993; Coslett *et al.*, 1995; Stark *et al.*, 1997; Huberle *et al.*, 2010; Thomas *et al.*, 2012; Shakespeare *et al.*, 2013) and letter-by-letter reading (Freedman *et al.*, 1991; Price and Humphreys, 1995). Therefore a sound understanding of lower-order oculomotor function in these patients is critical for the accurate interpretation of such visuoperceptual and other impairments in patients with PCA. Particularly given previous suggestions of impaired disengagement from fixation targets in PCA, this population also offers the opportunity to disambiguate between rival explanations of the normal gap/overlap effect (the lag in generating a saccade to a new target when the current fixation target persists), which has variously been attributed to parietal attentional disengagement and superior collicular active fixation mechanisms (Csibra *et al.*, 1997).

There are also strong anatomical and clinical rationales for investigating oculomotor function in PCA. Disruption of the parietal lobes—a primary site of atrophy in PCA (Whitwell *et al.*, 2007; Lehmann *et al.*, 2011*b*)—results in impaired saccadic latency, and deficits in smooth pursuit (Bogousslavsky and Regli, 1986; Pierrot-Deseilligny *et al.*, 1991; Braun *et al.*, 1992). Furthermore, disruption of the frontal eye fields—noted to be hypometabolic in PCA (Nestor *et al.*, 2003)—also results in oculomotor deficits such as increased saccadic latency, hypometric saccades, and impairment of smooth pursuit (Pierrot-Deseilligny *et al.*, 1997). Thus one would expect to find significant and frequent oculomotor abnormalities in patients with PCA, in contrast to the current low estimates derived from rudimentary clinical evaluations in the literature. It should be noted that in the current study, we deliberately restricted our investigations to pro- and not anti-saccade tasks in order to minimize attentional and inhibitory task demands and to focus on the role of the parietal eye fields in reflexive as opposed to intentional eye movements (Pa *et al.*, 2014).

In the current study we conducted the first systematic analysis of eye movement abnormalities in patients with PCA compared to patients with typical Alzheimer’s disease and healthy controls to address the central question of what impact oculomotor function has on higher-order perception in this syndrome. First we generated, compared and contrasted oculomotor profiles for each group across tests of fixation stability, saccade generation and smooth (sinusoidal) pursuit. Second we specifically examined patients’ ability to disengage attention and generate targets for subsequent eye movements using a saccade gap/overlap paradigm. Third we evaluated the relationship between metrics of oculomotor function and performance on tests of basic visual function and higher-order object and space perception. Fourth we investigated the structural neural correlates of fixation, saccade and pursuit abilities in PCA.

## Materials and methods

### Participants

#### Patient demographics

Data were collected from 20 patients with PCA (eight male), 17 patients with typical Alzheimer’s disease (nine male) and 22 healthy controls (five male). Patients with PCA fulfilled standard clinical criteria for PCA (Mendez *et al.*, 2002; Tang-Wai *et al.*, 2004) and had a clinical diagnosis of probable Alzheimer’s disease (Dubois *et al.*, 2007, 2010). Patients with typical Alzheimer’s disease fulfilled Dubois criteria for Alzheimer’s disease. At testing, 12 patients with typical Alzheimer’s disease and 13 patients with PCA were receiving treatment with acetyl-cholinesterase inhibitors. One patient with typical Alzheimer’s disease was receiving treatment with an NMDA (*N*-methyl D-aspartate) antagonist, and one patient with typical Alzheimer’s disease was receiving combined treatment with an acetyl-cholinesterase inhibitor and NMDA antagonist. Seven patients with typical Alzheimer’s disease and one with PCA were receiving treatment with antidepressants.

All participants completed the fixation task. Four patients with PCA did not complete the saccade task and two patients with PCA completed only the first two blocks of the saccade task, with four of these patients not completing the pursuit task due to fatigue. Five patients with typical Alzheimer’s disease did not complete the saccade task, one patient with typical Alzheimer’s disease did not complete the pursuit task. This project was approved by the NRES Committee London, Queen Square, and all participants provided written informed consent according to guidelines established by the Declaration of Helsinki.

#### Clinical presentation

In the PCA group, with the exception of one patient in whom the first noted symptom was repeating questions (closely followed by visuospatial symptoms), all patients with PCA reported their initial symptoms to be visuospatial, visuoperceptual or calculation difficulties (neuropsychological test scores of patients with PCA are presented in Supplementary Table 1). In the typical Alzheimer’s disease group, 15 patients reported episodic memory problems as their first symptom, one patient had word-finding difficulties followed by episodic memory and one patient had difficulties in navigation closely followed by episodic memory. Three of the patients with PCA had an amyloid (florbetapir) PET scan, performed as part of another study. Seven patients with PCA and 11 with typical Alzheimer’s disease had undergone CSF examination with measurement of amyloid-β_1-42_ and tau as part of their diagnostic evaluation.

Eighteen of 20 patients with PCA underwent neurological assessment performed by trained clinicians with expertise in the field of dementia (mean time interval between neurological and computerized eye movement assessment = 4.1 months). Oculomotor function was examined clinically with respect to the range of eye movements, subjective quality of pursuit movements and saccadic accuracy and speed. Any abnormality noted was qualified with a free text description of the deficit.

### Equipment

Stimuli were presented on a Dell Inspiron One desktop computer from a fixed viewing distance of 60 cm. Eye movements were recorded using a head-mounted infrared video-based eye tracker (Eyelink II; SR Research). Gaze position was recorded at 250 Hz and corneal reflection was used when possible (*n* = 8 PCA, *n* = 5 typical Alzheimer’s disease, *n* = 13 healthy controls). Participants used a chin rest (wide HeadSpot; University of Houston College of Optometry) to provide stability and maintain viewing distance throughout the experiment. Saccades were parsed by the Eyelink system, using standard velocity and acceleration thresholds (30°/s and 8000°/s^2^). Periods during which no saccadic movement occurred were automatically identified as fixation periods. We used built-in programs provided with the eye tracker for calibration and validation purposes (five points presented in a random sequence). All the data analysed were obtained from recordings with an average Cartesian prediction error of <1° during the validation procedures. Calibration was repeated before the start of a new task if participants needed a break from the eye tracker between tasks, or if there was slippage of the eye tracker between tasks. Each trial was initiated by a single target presented at the centre of the display (drift correct stimulus; grey inner circle (0.1°) with black outer circle (subtending 0.4° of visual angle). When the participant was fixating the target the experimenter initiated the trial, and any discrepancy between gaze location and the target location was corrected.

### Procedure

Testing took place in a quiet darkened room. All stimuli were presented on the display with a mid-grey background (RGB 128,128,128). The experimenter conducted experimental procedures positioned outside the participants’ field of view.

#### Fixation stability

Following the centrally presented drift correct stimulus, a red cross (RGB 255,0,0) subtending 0.5° of visual angle was presented. Participants were given one practice trial followed by a further three trials, in each trial the stimulus was presented for 10 s (following Crossland and Rubin, 2002). Participants were instructed to ‘look as closely as you can at the red cross without blinking for 10 seconds’.

#### Saccade assessment (gap and overlap conditions)

In the saccade task participants initially fixated a centrally presented stimulus, and were instructed to ‘look as quickly and accurately as you can to the new dot when it appears’ (following Garbutt *et al.*, 2008). The central fixation point was always presented for 500 ms. There were two target conditions:

- Gap condition: in half the trials target onset occurred 200 ms after fixation offset, so that there was only ever one stimulus on the screen at a given time.
- Overlap condition: in the other half of trials, target onset occurred 200 ms prior to fixation offset, so that for that 200 ms period two stimuli were simultaneously present on the screen (Kapoula *et al.*, 2010; Crawford *et al.*, 2013; Yang *et al.*, 2013).

In both conditions, the central fixation point was a circle the same size as that used for the drift correction, but with a white inner circle. The target was a larger version (inner white circle diameter 0.25°, outer black circle diameter 0.75°). Once presented, the target remained on the display until a fixation of minimum 250 ms duration was made within 1.5° of visual angle of the centre of the target, or until 5000 ms from target onset. Target stimuli were presented at 5, 10 and 15° horizontally, and 5 and 10° vertically from the centre of the display, giving a total of 10 target locations. There were four trials at each target location, yielding a total of 40 trials. There were an equal number of targets at each location in the gap and overlap conditions. Trials were split into four equal blocks (*n* = 10 each), with target locations randomized and balanced within each block, and all 10 target locations used within each block (with no locations repeated). The gap/overlap condition was alternated in an ABBA block design.

The saccade experiment included an additional condition in which the target made a small ‘jittering’ movement around the centre of the target location. There were 10 trials in each block interleaved with the stationary target trials in a randomized order. These trials were not analysed for the present study in order to retain the focus on low-level control mechanisms and to maintain clarity.

#### Sinusoidal pursuit

Two practice trials (one horizontal, then one vertical) were followed by six trials of sinusoidal pursuit, three horizontal and three vertical. The pursuit target was a red (RGB 255,0,0) circle 0.5° of visual angle in diameter. The movement had a total amplitude of 20° (10° either side of the centre). The frequency of the sinusoidal target oscillation was set at 0.25 Hz and each trial lasted 10 s (2.5 cycles). Each trial was initiated by the experimenter after a short interval allowing participants to re-orient to a central fixation point, from which the movement started.

### Analysis

Statistical analysis was carried out using Stata (v12.1) for each of the metrics described below with group as the independent variable, and age included as a covariate of no interest.

#### Fixation stability

Data from the practice trial were discarded.

##### Number of square wave jerks

Square wave jerks were defined as a saccade of <2° in amplitude, taking the gaze away from the target position, followed within 300 ms by another saccade with an amplitude similar to the first (difference in amplitude between saccades <0.75°), which takes gaze back towards the target position (Leigh and Zee, 2006). The number of square wave jerks during the fixation period for each participant was counted using an algorithm implementing the above rules.

##### Number of large intrusive saccades

Saccades containing blinks were removed. The number of saccades greater than 2° in amplitude was counted for each participant (Bylsma *et al.*, 1995).

##### Longest period of fixation

The maximum period of fixation (length of time between saccades) over all three trials was recorded for each participant (Bylsma *et al.*, 1995).

#### Saccade assessment

For the analysis of saccadic metrics, the stimulus eccentricity and whether the trial was a gap or overlap trial were included as additional independent variables.

##### Time to first fixation upon target

The time between the onset of the target and the first fixation made within 2.5° of the target was compared between groups. Time to first fixation upon target was not normally distributed, so analysis was performed on a square-root transform, with values greater or less than the two standard deviations from the mean of each group removed after transformation.

##### Amplitude, latency and velocity of first major saccade

Saccade amplitude, velocity and latency were calculated for the first major saccade towards the target (similar to Garbutt *et al.*, 2008). This saccade was identified using a predetermined algorithm based on pilot work; any saccade that included a blink, started before the target appears, started more than 2.5° from the central fixation point, or was in the wrong direction (error in angle of more than 45°) was removed. The first saccade remaining for each trial was kept. A trial was discarded if the saccade for that trial was the sixth saccade or later. The percentage of trials removed as a result of this procedure was 6.5% in healthy controls, 7.9% in typical Alzheimer’s disease and 11.3% in PCA.

The error in saccade amplitude was calculated as the difference between the amplitude of the main saccade and the eccentricity of the target. A positive value represents overshoot (hypermetria) whereas a negative value represents undershoot (hypometria).

Saccade latency was calculated as the time between onset of the target and the start of the main saccade (identified automatically using the algorithm described above). The distribution of saccade latency was very skew, so analysis was performed on a square-root transform of latency, with values of saccade latency greater or less than the two standard deviations from the mean of each group removed after transformation.

Peak saccadic velocity is very closely related to saccade amplitude (Boghen *et al.*, 1974), thus if saccadic amplitude was reduced in a patient group, we would expect peak saccadic velocity to also be reduced. Therefore in the comparison of peak saccadic velocity between groups, saccade amplitude was included as a covariate of no interest so that differences in velocity could be analysed independently from differences in saccadic amplitude. Saccade amplitude and velocity were provided by the Eyelink software.

##### Number of saccades made

The number of saccades that were made after the target appeared, did not include a blink and were greater than 2° was counted.

#### Sinusoidal pursuit

In the analysis of sinusoidal pursuit, pursuit direction (horizontal versus vertical) was included as an additional independent variable.

##### Pursuit gain

Pursuit gain (the ratio of eye velocity to target velocity) was calculated using Stata (v12.1) for each measurement sample of the eye tracker using instantaneous estimates (provided by the Eyelink system) of stimulus and gaze velocity. Blinks, saccades and periods of pupil occlusion (and samples 50 ms either side of any of these) were removed; this resulted in removing 4% of samples in healthy controls, 5% in typical Alzheimer’s disease and 6% in PCA. Gain was calculated for the remaining period, and outliers were removed [due to the differing nature of this measurement, the method used to remove outliers for latencies (removing samples where gain was greater than two standard deviations from the mean of that participant’s group) removed too much of the distribution of gain values, resulting in a biased group comparison; thus outliers were removed by cutting off the tails of the distribution of gain, defined by visual inspection of histograms, at −1 and +2].

##### Number of saccades

The number of saccades of amplitude greater than 2° was counted (not including blinks).

#### Receiver operator characteristic analysis

The extent of separation between the two groups at the individual patient level (rather than just in terms of the group means and variances) was investigated using a receiver operator characteristic (ROC) analysis. Analysis was carried out for the classification of patients with PCA and typical Alzheimer’s disease for each oculomotor metric, choosing the cut-off that maximized the percentage of patients correctly classified (accuracy). An additional analysis was carried out for the differentiation of patients (both PCA and typical Alzheimer’s disease) from healthy controls.

#### Correlations between oculomotor metrics and perceptual abilities

Pearson pairwise correlations and *post hoc* Bonferroni corrections were calculated between subsets of oculomotor metrics (number of square wave jerks and large saccades on fixation, saccade time to target, major saccade amplitude error and latency, and pursuit gain) and neuropsychological test scores (basic visual processing: figure-ground discrimination, shape discrimination; visuospatial: dot counting, number location; visuoperceptual: object decision, fragmented letters; non-visual tasks: graded difficulty arithmetic, short recognition memory test for words). Detailed descriptions of each of these neuropsychological tests and a table of individual test scores is presented in the Supplementary material.

### Neuroimaging

Thirteen patients with PCA and seven patients with typical Alzheimer’s disease who completed the eye-tracking assessments also had volumetric MRI available. Scans from 25 healthy controls were used for comparison (these are separate from the eye tracking controls, as those participants did not receive scans). T_1_-weighted volumetric MRI brain scans were acquired on a 3.0 T Siemens TIM Trio scanner using a magnetization prepared rapid gradient echo sequence with a 28.2 cm field-of-view to provide 208 contiguous 1.1 mm thick slices. Cortical thickness measurements were made using FreeSurfer version 5.3.0 (http://surfer.nmr.mgh.harvard.edu/). The detailed procedure has been described and validated in previous publications (Dale *et al.*, 1999; Fischl and Dale, 2000). Two modifications to the standard FreeSurfer processing stream were made: a locally generated brain mask was used to improve skull stripping, and FreeSurfer ventricular segmentations were added to the white matter mask to improve cortical segmentation.

Cortical thickness values were extracted for 34 brain areas in the left and right hemisphere using FreeSurfer’s Desikan parcellation (Desikan *et al.*, 2006). These areas were grouped into five larger regions: central, frontal, parietal, temporal, and occipital (following Ryan *et al.*, 2014; Supplementary Table 2). Cerebellar grey matter volume was extracted using FreeSurfer’s automatic subcortical segmentation (Fischl *et al.*, 2002).

In statistical analysis, cortical thickness was the dependent variable for the cortical regions, and volume was the dependent variable for the cerebellar grey matter. Group was the independent variable. Age, gender and total intracranial volume were included as additional covariates for adjustment. Associations with task performance were assessed in a combined patient group (patients with PCA and typical Alzheimer’s disease) to increase power.

## Results

### Patient characteristics

In terms of age at assessment, the PCA group [63.2 (8.9)] did not differ significantly from the healthy control group [mean (standard deviation, SD) age = 63.3 (6.2) years; two-sample *t*-test *P* = 0.97] or the typical Alzheimer’s disease group [mean (SD) age = 67.4 (5.9) years; *P* = 0.11]. However the typical Alzheimer’s disease group was significantly older than the healthy control group (*P* = 0.047). As described in the ‘Analysis’ section, age was included as a covariate in statistical analyses.

The PCA and typical Alzheimer’s disease groups did not differ in terms of disease duration [PCA: 4.6 (2.0) years; typical Alzheimer’s disease: 5.1 (2.4) years; *P* = 0.44] or Mini-Mental State Examination score [PCA: 18.8 (4.5); typical Alzheimer’s disease: 20.1 (5.2); *P* = 0.41].

Biomarkers of molecular pathology were available in 10/20 PCA and 11/17 typical Alzheimer’s disease patients (Table 1). These were supportive of underlying Alzheimer’s disease pathology in 8/10 PCA cases, compatible with Alzheimer’s disease in one case, with one case atypical for Alzheimer’s disease. In the typical Alzheimer’s disease group CSF was supportive of underlying Alzheimer’s disease in 10/11 cases, and atypical in one case.

**Table 1:** Molecular pathology biomarkers in patients

Diagnosis	PCA	PCA	PCA	PCA	PCA	PCA	PCA	PCA	PCA	PCA
Amyloid ^18^F imaging	N/A	N/A	N/A	N/A	N/A	N/A	N/A	Positive	Positive	Positive
CSF total tau (pg/ml)	841	787	325	412	561	310	898	N/A	N/A	N/A
CSF Aβ_1-42_ (pg/ml)	264	297	177	402	451	488	702	N/A	N/A	N/A
CSF tau: Aβ ratio	3.19	2.65	1.84	1.02	1.24	0.64	1.28	N/A	N/A	N/A
Biomarker interpretation	+	+	+	+	+	+/−	−	+	+	+

**Diagnosis**	**tAD**	**tAD**	**tAD**	**tAD**	**tAD**	**tAD**	**tAD**	**tAD**	**tAD**	**tAD**	**tAD**

Amyloid ^18^F imaging	N/A	N/A	N/A	N/A	N/A	N/A	N/A	N/A	N/A	N/A	N/A
CSF total tau (pg/ml)	828	843	1099	600	800	371	289	466	>1200	2722	203
CSF Aβ_1-42_ (pg/ml)	125	129	195	125	297	245	280	298	452	528	511
CSF tau: Aβ ratio	6.62	6.53	5.64	4.80	2.69	1.51	1.03	1.56	2.65	5.16	0.40
Biomarker interpretation	+	+	+	+	+	+	+	+	+	+	−

+= Supportive of Alzheimer’s disease (either positive ^18^ F florbetapir amyloid scan or CSF amyloid-β_1-42_ < 550 pg/ml and tau:amyloid-β ratio > 1).

+/−= Compatible with Alzheimer’s disease (borderline level of CSF amyloidβ_1-42_ or ratio).

−= Atypical for Alzheimer’s disease.

Aβ = amyloid-β; tAD = typical Alzheimer’s disease; N/A = not available.

Eighteen patients with PCA underwent clinical assessment of eye movements. Of these, 6/18 (33%) were noted to have eye movement abnormalities. These were hypometric saccades (*n* = 2), slow saccades (*n* = 1), gaze impersistence (*n* = 1), broken smooth pursuit (*n* = 1) and head thrust (*n* = 1). Case reports including clinical examination of eye movements from two patients with PCA with biomarkers suggestive of Alzheimer’s disease are presented in the Supplementary material.

### Fixation stability

Mean and standard deviation performance metrics for the fixation stability task are given in Table 2. Effect sizes (Cohen’s *d*) are presented in Supplementary Table 3.

**Table 2:** Mean and standard deviation performance metrics for PCA, typical Alzheimer’s disease and control groups on the fixation stability, saccade and sinusoidal pursuit tasks

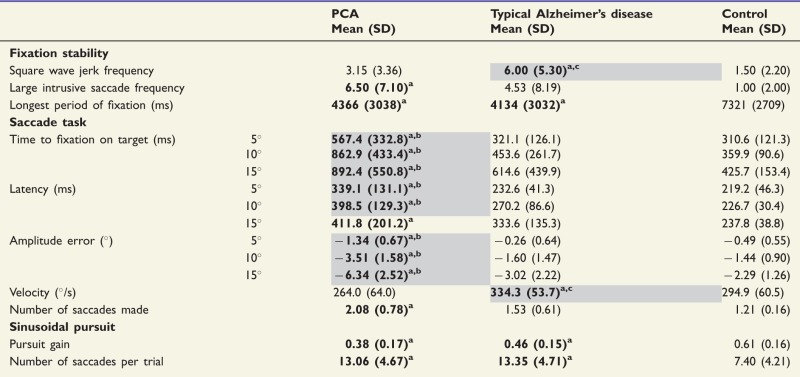

^a^Patient group performance significantly worse than controls (bold text).

^b^PCA group performance significantly worse than typical Alzheimer’s disease group performance (cells highlighted).

^c^Typical Alzheimer’s disease group performance significantly different from PCA group performance (cells highlighted).

All marked (^a,b,c^) significant comparisons indicate *P* ≤ 0.05; see text for exact significance values.

#### Square wave jerks

**Figure 1:**
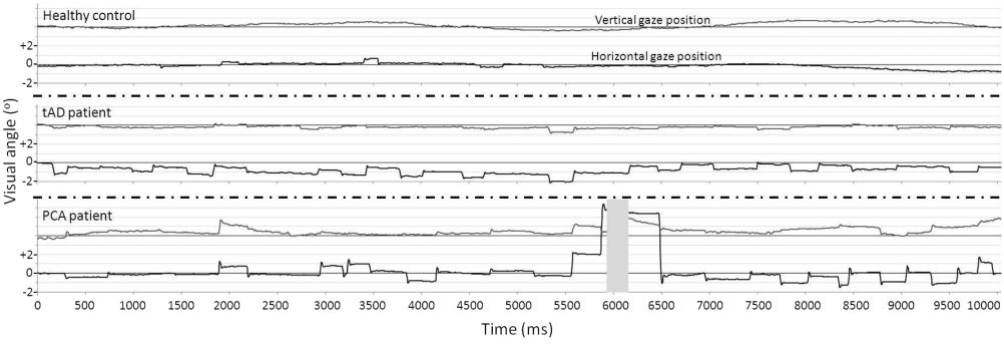
**Representative traces from the fixation task in a healthy control, a patient with typical Alzheimer’s disease and a PCA patient.** The *upper* plot (grey line) for each participant shows gaze position in the *y* (vertical) axis, the *lower* plot (black line) shows gaze position in the *x* (horizontal) axis. The location of the target stimulus is represented by thin black lines behind the traces. Gridlines show displacement of 1° of visual angle. The grey area in the plot for the PCA patient represents a blink (therefore *x* and *y* gaze coordinates are not available for this period). Positive values of gaze position indicate rightward gaze. The healthy control maintains steady fixation upon the target, whilst both patients show saccadic intrusions in the form of square-wave jerks. Additional large saccadic intrusions are evident in the PCA trace. tAD = typical Alzheimer’s disease.

Position traces for one participant from each group (control, typical Alzheimer’s disease, PCA) are shown in Fig. 1 to illustrate differences in the frequency of square wave jerks. The frequency of square wave jerks per trial did not differ significantly between patients with PCA and healthy controls [1.50 (2.20); *P* = 0.16]. A higher frequency of square wave jerks was observed in the patients with typical Alzheimer’s disease than patients with PCA (*P* = 0.02) or healthy controls (*P* < 0.001).

#### Number of large intrusive saccades

Patients with PCA showed a greater frequency of large intrusive saccades than healthy controls (*P* = 0.006), but did not differ significantly from patients with typical Alzheimer’s disease (*P* = 0.41). There was only a trend towards a greater frequency of large saccades in patients with typical Alzheimer’s disease than healthy controls (*P* = 0.07).

#### Longest period of fixation

The longest period of fixation was shorter in the PCA group than the control group (*P* = 0.002) but did not differ from the typical Alzheimer’s disease group (*P* = 0.81). Patients with typical Alzheimer’s disease showed a shorter maximum fixation period than the healthy controls (*P* = 0.002).

### Saccade assessment

**Figure 2:**
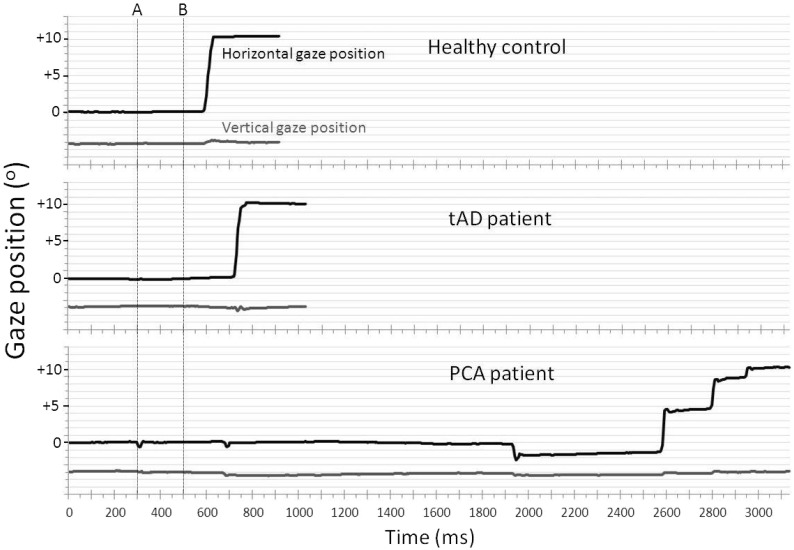
**Representative traces from the saccade task for a healthy control, a patient with typical Alzheimer’s disease and a PCA patient in an ‘overlap’ trial.** The *upper* plot (grey line) for each participant shows gaze position in the *y* (vertical) axis, the *lower* plot (black line) shows gaze position in the *x* (horizontal) axis. Gridlines show displacement of 1° of visual angle. Positive values of gaze position indicate rightward gaze. A central fixation point was present from the start of the trial until time point B (500 ms). The target appeared at 10° horizontally to the right of the central fixation point at time point A (300 ms) and remained present until the end of the trial. The healthy control and patients with typical Alzheimer’s disease make a single saccade towards the target. The PCA patient takes a long time to initiate their first saccade (in the incorrect direction), followed by a number of small saccades to reach the target location. tAD = typical Alzheimer’s disease.

Example traces from one participant from each group (control, typical Alzheimer’s disease, PCA) illustrating differences in their saccades, are presented in Fig. 2. Mean and standard deviation performance metrics for the saccade task are given in Table 2. Effect sizes (Cohen’s d) are presented in Supplementary Table 3.

#### Time to first fixation upon target

Patients with PCA took longer to reach the target at each eccentricity than patients with typical Alzheimer’s disease (5° *P* = 0.005, 10° *P* < 0.001, 15° *P* = 0.002) or controls (5° *P* < 0.001, 10° *P* < 0.001, 15° *P* < 0.001). Latencies in the typical Alzheimer’s disease group were not statistically different from those in controls at 5 or 10° of eccentricity, and there was only a trend at 15° (5° *P* = 0.28, 10° *P* = 0.21, 15° *P* = 0.07). This difference in time taken to fixate the target between patients with PCA and controls/patients with typical Alzheimer’s disease was greater at longer stimulus distances (interaction between group and distance *P* < 0.001).

##### Gap/overlap condition effect

**Figure 3:**
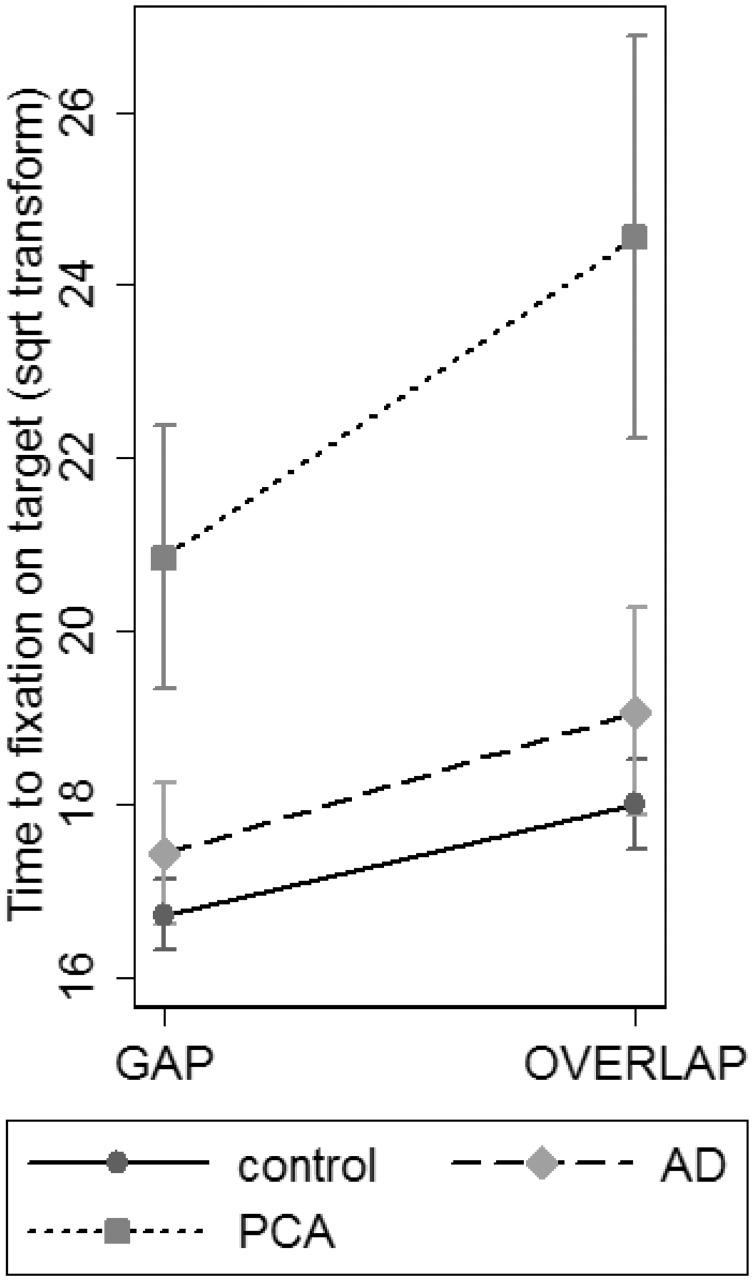
**Interaction figure showing greater effect of overlap condition on time taken to reach the interest area in patients with PCA relative to patients with typical Alzheimer’s disease and controls.** AD = Alzheimer’s disease.

There was also a significant interaction between group and the effect of the gap/overlap manipulation (group × gap/overlap interaction; *P* = 0.003; Fig. 3). *Post hoc* pairwise interaction analyses revealed that this main group × gap/overlap interaction reflected patients with PCA taking proportionally longer to reach the target in the overlap than gap condition compared with either patients with typical Alzheimer’s disease (*P* = 0.01) or controls (*P* = 0.01). By contrast there was no significant interaction between patients with typical Alzheimer’s disease and controls (*P* = 0.39).

#### Latency, amplitude and velocity of first major saccade

##### Saccade latency

Patients with PCA had longer latencies for the first major saccade towards the target than the control group at each target distance (5° *P* < 0.001, 10° *P* < 0.001, 15° *P* = 0.0497) and longer latencies than the typical Alzheimer’s disease group at 5° (*P* = 0.02) and 10° (*P* = 0.007), but not at 15° (*P* = 0.22). Latencies in the typical Alzheimer’s disease group were not statistically different from those in controls at any target distance (5° *P* = 0.27, 10° *P* = 0.29 and 15° *P* = 0.16). There was no statistically significant interaction between group and target distance (i.e. the difference in saccade latencies between groups was similar for each target distance; *P* = 0.67).

##### Saccade amplitude error

The main saccade towards the target was hypometric rather than hypermetric on average in each participant group, for each target distance (5, 10 and 15°). PCA patients’ main saccades had a smaller amplitude at each target distance than either controls (5° *P* < 0.001, 10° *P* < 0.001, 15° *P* < 0.001) or patients with typical Alzheimer’s disease (5° *P* = 0.001, 10° *P* < 0.002, 15° *P* = 0.001). There were no significant differences between patients with typical Alzheimer’s disease and controls in saccade amplitude error at any of the target distances (5° *P* = 0.59, 10° *P* = 0.61, 15° *P* = 0.18). There was an interaction between the effect of group and target distance (*P* < 0.001), with the difference between the patients with PCA and patients with typical Alzheimer’s disease/controls increasing with increasing target distance.

##### Saccade velocity

Saccade velocity (once saccade amplitude was accounted for) did not differ significantly between the PCA and control groups (*P* = 0.33). However, patients with typical Alzheimer’s disease showed increased peak saccadic velocity compared to both patients with PCA (*P* = 0.02) and healthy controls (*P* = 0.005).

#### Number of saccades made

Patients with PCA made more saccades per trial than healthy controls (*P* < 0.001) but showed only a trend towards more saccades than patients with typical Alzheimer’s disease (*P* = 0.07). Patients with typical Alzheimer’s disease only showed a trend towards more saccades than healthy controls (*P* = 0.06).

### Sinusoidal pursuit

**Figure 4:**
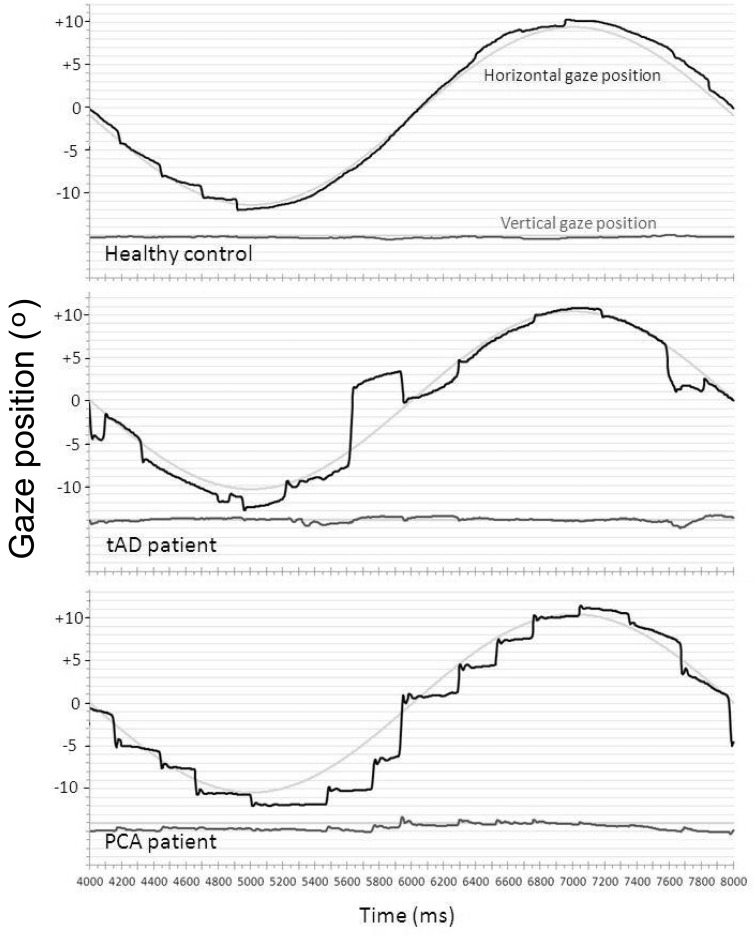
**Example traces from the pursuit task for a healthy control, a patient with typical Alzheimer’s disease and a PCA patient**. The figure shows a cycle towards the middle of the trial (seconds 4–8 from a trial of 10 s). Positive values of gaze position indicate rightward gaze. The *upper* plot (grey line) for each participant shows gaze position in the *y* (vertical) axis, the *lower* plot (black line) shows gaze position in the *x* (horizontal) axis. Target position is represented by a faint blue line. Gridlines show displacement of 1° of visual angle. tAD = typical Alzheimer’s disease.

Example traces from three participants illustrating performance in smooth pursuit are shown in Fig. 4. Mean and standard deviation performance metrics for the sinusoidal pursuit task are given in Table 2. Effect sizes (Cohen’s *d*) are presented in Supplementary Table 3.

#### Pursuit gain

Mean pursuit gain was significantly lower in the PCA group than the healthy control group (*P* < 0.001), but only a trend towards lower gain than the typical Alzheimer’s disease group (*P* = 0.09). The typical Alzheimer’s disease group also showed significantly lower gain than the control group (*P* = 0.01). Whilst participants showed lower gain for vertical compared to horizontal pursuit (mean difference in gain of 0.23 between conditions; *P* < 0.001), this effect was similar between patient groups (no interaction between group and pursuit direction; *P* = 0.93).

#### Number of saccades

Patients with PCA made more saccades per trial than healthy controls (*P* < 0.001), but did not differ from patients with typical Alzheimer’s disease (*P* = 0.94). Typical Alzheimer’s disease patients also made more saccades per trial than controls (*P* < 0.001).

#### Rate of oculomotor impairment and receiver operator characteristic analysis

Performance at the level of individual participants revealed a large separation between groups. Impaired performance was classed as a score more than 2 SD worse than healthy control performance. We looked at the proportion of patients and controls with impairments in more than one metric of saccadic performance: 12/15 (80%) of patients with PCA were impaired on more than one metric compared with only 2/12 (17%) of patients with typical Alzheimer’s disease and 1/22 (5%) of healthy controls.

In the receiver operator characteristic analysis, the metric with the greatest classification accuracy in the discrimination between PCA and typical Alzheimer’s disease was saccade amplitude error, which had a sensitivity of 93.8% and a specificity of 83.3%. Accuracy was higher for metrics of saccade performance than metrics of fixation and pursuit performance; the results for the remaining metrics are shown in Supplementary Table 4. The results from the receiver operator characteristic analysis for the discrimination of a combined patient group (PCA and typical Alzheimer’s disease) from healthy controls are presented in Supplementary Table 5.

### Association between oculomotor metrics and perceptual abilities

In the PCA group, time to saccadic target and major saccade latency each correlated significantly with almost all six basic visual, visuospatial and visuoperceptual tasks (*P* < 0.05 for 10/12 comparisons) but not with calculation or recognition memory scores (*P* > 0.05). None of the fixation or pursuit metrics correlated significantly with perceptual scores. The only comparison to survive Bonferroni corrections was the negative correlation between greater major saccade latency and poorer basic visual processing (shape discrimination test, r = 0.87, *P* < 0.001). In the typical Alzheimer’s disease group, there were significant pairwise correlations between time to saccadic target and figure-ground discrimination, pursuit gain and shape discrimination, and number of square wave jerks and calculation score. None of the typical Alzheimer’s disease correlations survived Bonferroni correction.

### Neuroimaging

**Figure 5:**
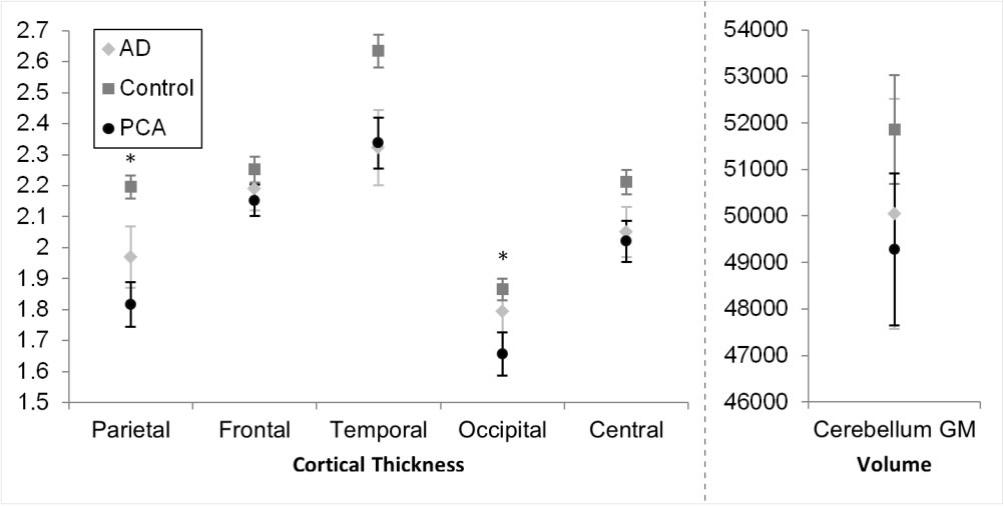
**Mean cortical thickness and cerebellar grey matter volume for each patient group in each region of interest.** Error bars indicate 95% confidence intervals. Asterisks indicate significant difference between patient groups. AD = Alzheimer’s disease; GM = grey matter.

*P*-values are provided in Table 3, and means and confidence intervals are presented in Fig. 5. Patients with PCA had lower cortical thickness than patients with typical Alzheimer’s disease and healthy controls in the parietal and occipital lobes. In the frontal, temporal and central regions, both patient groups had lower cortical thickness than controls, but did not differ from one another. In the cerebellar grey matter, patients with PCA had significantly lower volume than healthy controls, but did not differ significantly from patients with typical Alzheimer’s disease. Patients with typical Alzheimer’s disease did not differ significantly from healthy controls.

**Table 3:** *P*-values in the analysis of group differences in cortical thickness in each region of interest (upper section), and for correlations of oculomotor metrics with cortical thickness (combining both patient groups, but controls excluded; lower section)

Table of *P-*values	Parietal thickness	Frontal thickness	Temporal thickness	Occipital thickness	Central thickness	Cerebellar GM volume
Group differences						
Con versus PCA	**<0.001**	**0.005**	**<0.001**	**<0.001**	**<0.001**	**0.013**
Con versus tAD	**<0.001**	**0.041**	**<0.001**	0.072	**0.005**	0.205
PCA versus tAD	**0.043**	0.790	0.837	**0.022**	0.341	0.634
Correlations in combined patient group						
SWJs in fixation	0.624	0.152	0.980	0.667	0.500	**0.003**
Int. sac. in fix^a^	**0.005**	**0.002**	**0.036**	**<0.001**	0.111	0.801
Saccade time to target	**0.027**	0.939	0.357	**0.037**	0.170	0.623
Saccade amplitude error	0.336	0.703	0.600	0.057	0.478	0.993
Velocity gain (pursuit)	0.409	0.849	0.995	0.115	0.881	0.194

Bold highlight indicates significant effects.

^a^Frequency of intrusive saccades during fixation.

Con = control; GM = grey matter; tAD = typical Alzheimer’s disease; SWJ = square wave jerk.

In the combined patient group (patients with PCA and typical Alzheimer’s disease) there was a significant negative correlation between a greater frequency of square wave jerks during fixation and lower cerebellar grey matter volume, but not with any cortical area. By contrast, large saccadic intrusion frequency was associated with reductions in cortical thickness metrics but not cerebellar grey matter volume. There was a significant association between longer time to reach the saccade target and lower cortical thickness in the parietal lobe and the occipital lobe, but no other regions of interest. Major saccade amplitude and velocity gain during smooth pursuit did not correlate significantly with cortical thickness in any of the cortical regions, or with cerebellar grey matter volume.

## Discussion

We describe the first detailed assessment of oculomotor function in patients with PCA as compared with patients with typical Alzheimer’s disease and healthy controls. In patients with PCA, the most prominent deficits (where they were significantly worse than not only controls but also patients with typical Alzheimer’s disease) were in increased time to saccadic target fixation, increased first major saccade latency and decreased saccade amplitude. The patients with PCA showed additional deficits (relative to controls) on fixation stability (more frequent large intrusive saccades and lower longest period of fixation) and sinusoidal pursuit (lower pursuit gain and more saccades per trial). However, the peak velocity of saccades was normal (after accounting for saccade amplitude) indicating relative preservation of motor aspects of eye movement generation.

By contrast, the pattern of oculomotor dysfunction in the typical Alzheimer’s disease group was characterized (relative to both patients with PCA and controls) by more square wave jerks during fixation and increased saccadic velocity, and showed additional deficits (relative to controls) of lower longest period of fixation, lower pursuit gain and more saccades per pursuit trial. However, unlike patients with PCA, patients with typical Alzheimer’s disease did not differ from controls on any of the saccade task measures (i.e. normal time to target, major saccade latency and amplitude, and number of saccades) except their increased saccade velocity.

Thus patients with PCA showed deficits across all three fixation, saccade and pursuit tasks with deficits particularly evident on the saccade task (especially in the overlap condition), whereas typical Alzheimer’s disease patients’ oculomotor deficits were largely confined to fixation and pursuit deficits. The overall greater oculomotor impairment of the PCA relative to typical Alzheimer’s disease group occurred despite the fact that the patients with PCA were younger than the patients with typical Alzheimer’s disease (note that age was included as a covariate in statistical analyses) and the patient groups were matched for Mini-Mental State Examination and disease duration. Here we consider the theoretical, clinical and anatomical implications of PCA and typical Alzheimer’s disease performance with regard to each of the three eye movement behaviours (fixation, saccade and pursuit) in turn.

### Fixation stability

The findings of impaired fixation stability in PCA and typical Alzheimer’s disease warrant further discussion. Both groups exhibited a decreased period of fixation, which was associated in PCA with increased frequency of large intrusive saccades and in typical Alzheimer’s disease with an increased frequency of square wave jerks. These findings of impaired fixation stability are consistent with previous reports of saccadic intrusions during fixation in Alzheimer’s disease (Jones *et al.*, 1983; Schewe *et al.*, 1999), though it should be noted that square wave jerks are not specific to Alzheimer’s disease, with increased frequency associated with advancing age (Herishanu and Sharpe, 1981) and other neurological conditions such as progressive supranuclear palsy, cerebellar disease and Parkinson’s disease (Troost and Daroff, 1977; White *et al.*, 1983; Rascol *et al.*, 1991; Rabiah *et al.*, 1997). The increased frequency of square wave jerks in patients with typical Alzheimer’s disease likely reflects changes in basic oculomotor mechanisms rather than higher order cognitive processes, as these eye movements are not under cognitive control. Our imaging analyses are consistent with this interpretation given the observed association between square wave jerk frequency and cerebellar grey matter volume but none of the indices of cortical thickness.

Of particular interest in the PCA data was not only the identification of large saccadic intrusions but also the fact that these large intrusions occurred with greater frequency than smaller square wave jerks. This suggests that these large saccadic intrusions may have a different origin, perhaps associated with the visual disorientation or visual inattention that commonly accompany this syndrome. Again, the imaging data are consistent with this interpretation; large saccadic intrusion frequency in the patients with PCA was significantly associated with reductions in cortical thickness metrics but not (as in the case of square wave jerks) with cerebellar grey matter volume.

Turning to the clinical impact of these fixation abnormalities, square wave jerks of the kind measured here in typical Alzheimer’s disease and PCA are not generally considered to have strongly adverse effects on visual perception. By contrast, these larger saccadic intrusions that shift gaze to a new location and do not involve a rapid return to the target mean that the individual may completely lose track of the target they are trying to monitor. Indeed, it has previously been proposed in a single case study, that aberrant involuntary saccadic eye movements may underlie some PCA patients’ experience of apparent motion amongst static objects (e.g. letters in a words, or dots on a page, appear to be moving; Crutch *et al.*, 2011). What remains unclear is whether the major saccade away from the target is an involuntary movement, or whether it reflects the beginning of an erroneous voluntary search process triggered by a minor involuntary movement that, whilst small, is sufficient to give patients with PCA the impression that they have lost the target. We therefore suggest these large intrusions may have two components, both of which are impaired in PCA: an initial intrusion (small or large, attributable to basic oculomotor dysfunction), and an impaired refixation process (attributable to basic and higher-order perceptual and/or attentional impairments).

### Saccades: a deficit of target generation or movement generation?

Previous studies of PCA note the occurrence of oculomotor apraxia (also known as ocular apraxia; a reduced ability to make voluntary saccades) on informal clinical testing in an average of 28% of patients with PCA (consistent with the 33% on clinical assessment in the present study), but the proportion varies considerably between studies [10% (Tang-Wai *et al.*, 2004), 27% (Mendez *et al.*, 2002), 38% (Kas *et al.*, 2011), 47% (McMonagle *et al.*, 2006)]. By contrast, our detailed quantitative examination of eye movements found evidence of oculomotor impairment in 80% of patients with PCA (relative to 17% typical Alzheimer’s disease and 5% controls), a level between 2 and 8 times greater than those previous clinical estimates. The receiver operator characteristic analysis also revealed a high degree of separation between PCA and typical Alzheimer’s disease groups for saccade amplitude error (sensitivity 93.8%, specificity 83.3%). Therefore these data demonstrate that oculomotor deficits are present in the vast majority of individuals with PCA and should be regarded as a core feature of the PCA syndrome. Whilst we do not propose that this technique should be used in isolation as a diagnostic test, the results suggest sensitive tests of oculomotor function are also informative at the individual level. More broadly, these data add to the growing evidence that investigating eye movements may be helpful in identifying and differentiating a number of neurodegenerative conditions (Garbutt *et al.*, 2008; Boxer *et al.*, 2012).

Furthermore we found a significantly greater effect of the gap/overlap manipulation upon time to first fixation upon the target in the PCA group compared to the typical Alzheimer’s disease and healthy control groups. The normal gap/overlap effect has been attributed to a generalized warning signal effect arising from the offset of the old target (Csibra *et al.*, 1997; Abel and Yee, 2002), parietal-driven disengagement of visual attention from the attended stimulus (Posner *et al.*, 1984; Fischer and Breitmeyer, 1987; Fischer and Weber, 1993; Csibra *et al.*, 1997) and a termination of activity by an active fixation mechanism in the superior colliculus (Reuter-Lorenz *et al.*, 1991; Kingstone and Klein, 1993; Tam and Stelmach, 1993; Klein *et al.*, 1995). The significantly exacerbated gap/overlap effect observed in patients with PCA may reflect poor attentional disengagement from the current focus of fixation. However, given the superior colliculus’ role in generating eye movements towards new target locations based on a 2D map of retinotopic space, in patients with PCA weak occipital and parietal input regarding target location may also lead to a slow and/or inaccurate build up of ‘hill’ activity within these superior collicular coordinates. This notion of weak input to the subcortical oculomotor system from degraded higher-order cortical spatial representations fits with the neuroimaging data that showed significant associations between time to reach the target and parietal and occipital cortical thickness measures. This notion is also consistent with significant correlations between the extent of disruption of PCA saccade metrics and the extent of their basic, visuospatial and visuoperceptual impairment in the background neuropsychological assessment of occipital and parietally-mediated cognitive functions. The finding of normal saccadic velocities in patients with PCA adds weight to the argument that PCA patients’ longer latencies to reach the target location reflect impairment of oculomotor target identification rather than the execution of oculomotor movements. Regarding the relevance of these findings to studies of visual salience and real world perception, it should be noted that to date PCA scene perception has only been evaluated using static scene photographs (Mannan *et al.*, 2009; Foulsham *et al.*, 2011; Shakespeare *et al.*, 2013). However, the current results regarding oculomotor responses to changes in an (albeit very simple) scene emphasize the critical role not only of spatial attention and object identification but also precise localization of features/changes in understanding how these individuals perceive the real world.

Briefly in reference to patients with typical Alzheimer’s disease, it should also be noted that in contrast to previous studies of pro-saccades in this population (Fletcher and Sharpe, 1986; Bylsma *et al.*, 1995; Yang *et al.*, 2011, 2013), we did not see any systematic differences between our typical Alzheimer’s disease group and the healthy control group, with the exception of increased peak saccadic velocity (after controlling for saccade amplitude). Furthermore, two previous studies have reported a normal gap/overlap effect (Abel and Yee, 2002; Crawford *et al.*, 2013) whilst one study reported an exaggerated effect (Yang *et al.*, 2013). The results from the present study add weight to the former position, as there was no difference in the magnitude of the gap/overlap effect exhibited by patients with typical Alzheimer’s disease and controls.

### Pursuit function

In the smooth pursuit measures, the PCA and typical Alzheimer’s disease groups did not differ from one another, with both groups showing lower gain than the healthy controls and an increased frequency of intrusive saccades (although there was a weak trend to lower pursuit gain in PCA than typical Alzheimer’s disease). This is consistent with previous studies of smooth pursuit in typical Alzheimer’s disease (Fletcher and Sharpe, 1988; Zaccara *et al.*, 1992; Garbutt *et al.*, 2008). The trace showing impaired smooth pursuit in PCA in Fig. 4 was chosen as an interesting example (this performance is not representative of the patients with PCA, the majority showed similar performance to patients with typical Alzheimer’s disease in this task); this pattern is indicative of basic oculomotor dysfunction; this PCA patient is clearly tracking the target (suggesting that the representation of target motion and planned gaze location is at least partially preserved), but does so with multiple saccades and very little smooth pursuit (suggesting an impairment in the oculomotor mechanism underlying smooth pursuit).

### Limitations and future directions

Finally, it is worth considering the potential weaknesses of this study. Whilst all the patients with PCA met clinical criteria for the syndrome, and did not exhibit symptoms suggestive of pathologies other than Alzheimer’s pathology (e.g. early hallucinations, delusions and fluctuations suggesting Lewy body disease), it remains possible that some of the participants do not have Alzheimer’s disease, or have coexistent pathologies (Renner *et al.*, 2004). The PCA and typical Alzheimer’s disease groups were not matched for age, with the patients with PCA being younger, as is typically the case in this syndrome which typically has age-at-onset in the sixth or seventh decade. However, we covaried for age in our statistical analyses, and given that the patients with PCA (who were younger) performed worse than patients with typical Alzheimer’s disease on many of the tasks, this suggests that if there is an effect of age it would result in an underestimate of the differences between the patients with PCA and typical Alzheimer’s disease. Furthermore not all participants had concurrent imaging data available; repeating the analyses conducted with larger sample sizes would increase sensitivity to detect associations between oculomotor behaviour and specific cortical subregions such as the parietal and frontal eye fields. The experimental design of the saccade task meant that the final trial of each block was theoretically predictable (given each location was tested in each block) and improved design would avoid this issue.

One further point (which we do not regard as a limitation) relates to the analysis method for saccadic latency, amplitude and velocity. Participants’ performance did not allow straightforward identification of the first or main saccade towards the target (e.g. making a number of small saccades near the fixation point before making a saccade towards the target, or making a number of smaller saccades towards the target). We therefore developed an algorithm to define the main saccade that is clear and reproducible. However, the combination of patient performance and choice of analysis method may mean that results of this algorithm are not interpretable in exactly the same way as a standard saccadometry experiment, but we do feel they accurately represent performance in this patient group.

We speculate that the increased frequency of large saccades during fixation in patients with PCA could be due to spontaneous motor activity; however, an alternative explanation would be greater distractibility or poorer inhibitory control in the patients with PCA. Although we cannot distinguish between these alternatives objectively on the basis of the data collected, the subjective experience while testing the PCA participants was of effortful performance rather than distractibility; however, it would be interesting to further investigate this aspect experimentally. One avenue of future research of interest is to investigate performance in an antisaccade task. This task requires participants to move their eyes in the opposite direction to a visually presented stimulus (Antoniades *et al.*, 2013) and is usually considered a marker of inhibitory control (Kaufman *et al.*, 2010; Crawford *et al.*, 2013). It is possible that this may help to discriminate between spontaneous motor activity versus distractibility or poor inhibitory control. However in patients with PCA, interpretation in this test may be clouded by their concomitant left-right disorientation, simultanagnosia and impairments in saccadic performance. Nonetheless examination of antisaccade performance and other oculomotor behaviours such as predictive saccades could expand the current investigation of low-level control mechanisms to higher-order aspects of oculomotor control.

Future studies might also capitalize on other imaging modalities (e.g. diffusion tensor imaging) to look at the integrity of subcortical structures and white matter tracts to test the stated hypotheses regarding the role of parietal-superior colliculus connections in determining oculomotor behaviour in PCA and their broader impact upon higher-order perception. Such data would build on the insights offered by the current study regarding the combined role of oculomotor dysfunction and higher-order perceptual impairment in explaining the experiences of people with PCA and the broader population of individuals with dementia-related visual dysfunction.

## Abbreviation

PCA: posterior cortical atrophy

## References

[awv103-B1] Abel LA, Yee RD (2002). Effects of stimulus predictability and interstimulus gap on saccades in Alzheimer’ s Disease.

[awv103-B2] Antoniades C, Ettinger U, Gaymard B, Gilchrist I, Kristjánsson A, Kennard C (2013). An internationally standardized antisaccade protocol. Vision Res.

[awv103-B3] Bak TH, Caine D, Hearn VC, Hodges JR (2006). Visuospatial functions in atypical parkinsonian syndromes. J Neurol Neurosurg Psychiatry.

[awv103-B4] Benson DF, Davis RJ, Snyder BD (1988). Posterior cortical atrophy. Arch Neurol.

[awv103-B5] Boghen D, Troost BT, Daroff RB, Dell’Osso LF, Birkett JE (1974). Velocity characteristics of normal human saccades. Invest Ophthalmol.

[awv103-B6] Bogousslavsky J, Regli F (1986). Pursuit gaze defects in acute and chronic unilateral parieto-occipital lesions. Eur Neurol.

[awv103-B7] Boxer AL, Garbutt S, Seeley WW, Jafari A, Heuer HW, Mirsky J (2012). Saccade abnormalities in autopsy-confirmed frontotemporal lobar degeneration and Alzheimer disease. Arch Neurol.

[awv103-B8] Braun D, Weber H, Mergner T, Schulte-Mönting J (1992). Saccadic reaction times in patients with frontal and parietal lesions. Brain.

[awv103-B9] Bylsma FW, Rasmusson DX, Rebok GW, Key PM, Tune L, Brandt J (1995). Changes in visual fixation and saccadic eye movements in Alzheimer’ s disease. Int J Psychophysiol.

[awv103-B10] Coslett HB, Stark M, Rajaram S, Saffran EM (1995). Narrowing the spotlight: a visual attentional disorder in presumed Alzheimer’s disease. Neurocase.

[awv103-B11] Crawford T, Higham S, Mayes J, Dale M, Shaunak S, Lekwuwa G (2013). The role of working memory and attentional disengagement on inhibitory control: effects of aging and Alzheimer’s disease. Age (Omaha).

[awv103-B12] Crossland MD, Rubin GS (2002). The use of an infrared eyetracker to measure fixation stability. Optom Vis Sci.

[awv103-B13] Crutch SJ, Lehmann M, Gorgoraptis N, Kaski D, Ryan N, Husain M (2011). Abnormal visual phenomena in posterior cortical atrophy. Neurocase.

[awv103-B14] Crutch SJ, Lehmann M, Schott JM, Rabinovici GD, Rossor MN, Fox NC (2012). Posterior cortical atrophy. Lancet Neurol.

[awv103-B15] Csibra G, Johnson MH, Tucker LA (1997). Attention and oculomotor control: a high-density ERP study of the gap effect. Neuropsychologia.

[awv103-B16] Dale AM, Fischl B, Sereno MI (1999). Cortical surface-based analysis. I. Segmentation and surface reconstruction. Neuroimage.

[awv103-B17] Delamont RS, Harrison J, Field M, Boyle RS (1989). Posterior cortical atrophy. Clin Exp Neurol.

[awv103-B18] Desikan RS, Ségonne F, Fischl B, Quinn BT, Dickerson BC, Blacker D (2006). An automated labeling system for subdividing the human cerebral cortex on MRI scans into gyral based regions of interest. Neuroimage.

[awv103-B19] Dubois B, Feldman HH, Jacova C, Cummings JL, Dekosky ST, Barberger-Gateau P (2010). Revising the definition of Alzheimer’s disease: a new lexicon. Lancet Neurol.

[awv103-B20] Dubois B, Feldman HH, Jacova C, Dekosky ST, Barberger-Gateau P, Cummings J (2007). Research criteria for the diagnosis of Alzheimer’s disease: revising the NINCDS-ADRDA criteria. Lancet Neurol.

[awv103-B21] Dubois B, Feldman HH, Jacova C, Hampel H, Molinuevo JL, Blennow K (2014). Advancing research diagnostic criteria for Alzheimer’s disease: the IWG-2 criteria. Lancet Neurol.

[awv103-B22] Fischer B, Breitmeyer B (1987). Mechanisms of visual attention revealed by saccadic eye movements. Neuropsychologia.

[awv103-B23] Fischer B, Weber H (1993). Express saccades and visual attention. Behav Brain Sci.

[awv103-B24] Fischl B, Dale AM (2000). Measuring the thickness of the human cerebral cortex from magnetic resonance images. Proc Natl Acad Sci USA.

[awv103-B25] Fischl B, Salat DH, Busa E, Albert M, Dieterich M, Haselgrove C (2002). Whole Brain segmentation: neurotechnique automated labeling of neuroanatomical structures in the human brain. Neuron.

[awv103-B26] Fletcher WA, Sharpe JA (1986). Saccadic eye movement dysfunction in Alzheimer’s disease. Ann Neurol.

[awv103-B27] Fletcher WA, Sharpe JA (1988). Smooth pursuit dysfunction in Alzheimer’s disease. Neurology.

[awv103-B28] Foulsham T, Barton JJS, Kingstone A, Dewhurst R, Underwood G (2011). Modeling eye movements in visual agnosia with a saliency map approach: bottom-up guidance or top-down strategy?. Neural networks.

[awv103-B29] Freedman L, Selchen DH, Black SE, Kaplan R, Garnett ES, Nahmias C (1991). Posterior cortical dementia with alexia: neurobehavioural, MRI, and PET findings. J Neurol Neurosurg Psychiatry.

[awv103-B30] Garbutt S, Matlin A, Hellmuth J, Schenk AK, Johnson JK, Rosen H (2008). Oculomotor function in frontotemporal lobar degeneration, related disorders and Alzheimer’s disease. Brain.

[awv103-B31] Golding CVP, Danchaivijitr C, Hodgson TL, Tabrizi SJ, Kennard C (2006). Identification of an oculomotor biomarker of preclinical Huntington disease. Neurology.

[awv103-B32] Graff-Radford NR, Bolling JP, Earnest F, Shuster EA, Caselli RJ, Brazis PW (1993). Simultanagnosia as the initial sign of degenerative dementia. Mayo Clin Proc.

[awv103-B33] Herishanu YO, Sharpe JA (1981). Normal square wave jerks. Invest Ophthalmol Vis Sci.

[awv103-B34] Hicks SL, Robert MP, Golding CVP, Tabrizi SJ, Kennard C (2008). Oculomotor deficits indicate the progression of Huntington’s disease. Prog Brain Res.

[awv103-B35] Hof PR, Bouras C, Constantinidis J, Morrison JH (1990). Selective disconnection of specific visual association pathways in cases of Alzheimer’s disease presenting with Balint's syndrome. J Neuropathol Exp Neurol.

[awv103-B36] Huberle E, Driver J, Karnath H-O (2010). Retinal versus physical stimulus size as determinants of visual perception in simultanagnosia. Neuropsychologia.

[awv103-B37] Jones A, Friedland RP, Koss B, Stark L, Thompkins-Ober BA (1983). Saccadic intrusions in Alzheimer-type dementia. J Neurol.

[awv103-B38] Kapoula Z, Yang Q, Vernet M, Dieudonné B, Greffard S, Verny M (2010). Spread deficits in initiation, speed and accuracy of horizontal and vertical automatic saccades in dementia with lewy bodies. Front Neurol.

[awv103-B39] Kaufman LD, Pratt J, Levine B, Black SE (2010). Antisaccades: a probe into the dorsolateral prefrontal cortex in Alzheimer’s disease: a critical review. J Alzheimers Dis.

[awv103-B40] Kas A, de Souza LC, Samri D, Bartolomeo P, Lacomblez L, Kalafat M (2011). Neural correlates of cognitive impairment in posterior cortical atrophy. Brain.

[awv103-B41] Kingstone A, Klein RM (1993). Visual offsets facilitate saccadic latency: does predisengagement of visuospatial attention mediate this gap effect?. J Exp Psychol Hum Percept Perform.

[awv103-B42] Klein RM, Taylor TL, Kingstone A (1995). Against a role for attentional disengagement in the gap effect: a friendly amendment to Tam and Stelmach (1993). Percept Psychophys.

[awv103-B43] Kouri N, Whitwell JL, Josephs KA, Rademakers R, Dickson DW (2011). Corticobasal degeneration: a pathologically distinct 4R tauopathy. Nat Rev Neurol.

[awv103-B44] Lehmann M, Barnes J, Ridgway GR, Wattam-Bell J, Warrington EK, Fox NC (2011a). Basic visual function and cortical thickness patterns in posterior cortical atrophy. Cereb Cortex.

[awv103-B45] Lehmann M, Crutch SJ, Ridgway GR, Ridha BH, Barnes J, Warrington EK (2011b). Cortical thickness and voxel-based morphometry in posterior cortical atrophy and typical Alzheimer’s disease. Neurobiol Aging.

[awv103-B46] Leigh RJ, Zee DS (2006). The neurology of eye movements.

[awv103-B47] Mannan SK, Kennard C, Husain M (2009). The role of visual salience in directing eye movements in visual object agnosia. Curr Biol.

[awv103-B48] McKhann GM, Knopman DS, Chertkow H, Hyman BT, Jack CR, Kawas CH (2011). The diagnosis of dementia due to Alzheimer’s disease: recommendations from the National Institute on Aging-Alzheimer's Association workgroups on diagnostic guidelines for Alzheimer's disease. Alzheimers Dement.

[awv103-B49] McMonagle P, Deering F, Berliner Y, Kertesz A (2006). The cognitive profile of posterior cortical atrophy. Neurology.

[awv103-B50] Mendez M, Ghajarania M, Perryman KM (2002). Posterior cortical atrophy: clinical characteristics and differences compared to Alzheimer’s disease. Dement Geriatr Cogn Disord.

[awv103-B51] Nestor PJ, Caine D, Fryer TD, Clarke J, Hodges JR (2003). The topography of metabolic deficits in posterior cortical atrophy (the visual variant of Alzheimer’s disease) with FDG-PET. J Neurol Neurosurg Psychiatry.

[awv103-B52] Pa J, Dutt S, Mirsky JB, Heuer HW, Keselman P, Kong E (2014). The functional oculomotor network and saccadic cognitive control in healthy elders. Neuroimage.

[awv103-B53] Pierrot-Deseilligny C, Gaymard B, Müri R, Rivaud S (1997). Cerebral ocular motor signs. J Neurol.

[awv103-B54] Pierrot-Deseilligny C, Rivaud S, Gaymard B, Agid Y (1991). Cortical control of reflexive visually-guided saccades. Brain.

[awv103-B55] Posner MI, Walker JA, Friedrich FJ, Rafal RD (1984). Effects of parietal injury on covert orienting of attention. J Neurosci.

[awv103-B56] Price CJ, Humphreys GW (1995). Contrasting effects of letter-spacing in alexia: further evidence that different strategies generate word length effects in reading. Q J Exp Psychol A.

[awv103-B57] Rabiah PK, Bateman JB, Demer JL, Perlman S (1997). Ophthalmologic findings in patients with ataxia. Am J Ophthalmol.

[awv103-B58] Rascol O, Sabatini U, Simonetta-Moreau M, Montastruc JL, Rascol A, Clanet M (1991). Square wave jerks in parkinsonian syndromes. J. Neurol. Neurosurg. Psychiatry.

[awv103-B59] Renner JA, Burns JM, Hou CE, McKeel DW, Storandt M, Morris JC (2004). Progressive posterior cortical dysfunction: a clinicopathologic series. Neurology.

[awv103-B60] Reuter-Lorenz PA, Hughes HC, Fendrich R (1991). The reduction of saccadic latency by prior offset of the fixation point: an analysis of the gap effect. Percept Psychophys.

[awv103-B61] Rohrer JD, Paviour D, Bronstein AM, O’Sullivan SS, Lees A, Warren JD (2010). Progressive supranuclear palsy syndrome presenting as progressive nonfluent aphasia: a neuropsychological and neuroimaging analysis. Mov Disord.

[awv103-B62] Ryan NS, Shakespeare TJ, Lehmann M, Keihaninejad S, Nicholas JM, Leung KK (2014). Motor features in posterior cortical atrophy and their imaging correlates. Neurobiol Aging.

[awv103-B63] Schewe HJ, Uebelhack R, Vohs K (1999). Abnormality in saccadic eye movement in dementia. Eur Psychiatry.

[awv103-B64] Shakespeare TJ, Yong KXX, Frost C, Kim LG, Warrington EK, Crutch SJ (2013). Scene perception in posterior cortical atrophy: categorization, description and fixation patterns. Front Hum Neurosci.

[awv103-B65] Sharma R, Hicks S, Berna CM, Kennard C, Talbot K, Turner MR (2011). Oculomotor dysfunction in amyotrophic lateral sclerosis: a comprehensive review. Arch Neurol.

[awv103-B66] Stark ME, Grafman J, Fertig E (1997). A restricted “spotlight” of attention in visual object recognition. Neuropsychologia.

[awv103-B67] Tam WJ, Stelmach LB (1993). Viewing behavior: ocular and attentional disengagement. Percept Psychophys.

[awv103-B68] Tang-Wai DF, Graff-Radford NR, Boeve BF, Dickson DW, Parisi JE, Crook R (2004). Clinical, genetic, and neuropathologic characteristics of posterior cortical atrophy. Neurology.

[awv103-B69] Tang-Wai DF, Josephs KA, Boeve BF, Dickson DW, Parisi JE, Petersen RC (2003). Pathologically confirmed corticobasal degeneration presenting with visuospatial dysfunction. Neurology.

[awv103-B70] Thomas C, Kveraga K, Huberle E, Karnath H-O, Bar M (2012). Enabling global processing in simultanagnosia by psychophysical biasing of visual pathways. Brain.

[awv103-B71] Troost BT, Daroff RB (1977). The ocular motor defects in progressive supranuclear palsy. Ann Neurol.

[awv103-B72] White S-CJ, Tomlinson R, Sharpe J (1983). Ocular motor deficits in Parkinson’s disease II. Control of the saccadic and smooth pursuit systems. Brain.

[awv103-B73] Whitwell JL, Jack CR, Kantarci K, Weigand SD, Boeve BF, Knopman DS (2007). Imaging correlates of posterior cortical atrophy. Neurobiol Aging.

[awv103-B74] Yang Q, Wang T, Su N, Liu Y, Xiao S, Kapoula Z (2011). Long latency and high variability in accuracy-speed of prosaccades in Alzheimer’s disease at mild to moderate stage. Dement Geriatr Cogn Dis Extra.

[awv103-B75] Yang Q, Wang T, Su N, Xiao S, Kapoula Z (2013). Specific saccade deficits in patients with Alzheimer’s disease at mild to moderate stage and in patients with amnestic mild cognitive impairment. Age (Omaha).

[awv103-B76] Yong K, Warrington EK, Shakespeare TJ, Cash D, Henley SMD, Warren JD (2014). Prominent effects and neural correlates of visual crowding in a neurodegenerative disease population. Brain.

[awv103-B77] Zaccara G, Gangemi PF, Muscas GC, Paganini M, Pallanti S, Parigi A (1992). Smooth-pursuit eye movements: alterations in Alzheimer’s disease. J Neurol Sci.

